# Siderol Inhibits Proliferation of Glioblastoma Cells and Acts Synergistically with Temozolomide

**DOI:** 10.3390/biomedicines10123216

**Published:** 2022-12-12

**Authors:** Maria Giannakopoulou, Kiriakos Dimitriadis, Maria Koromili, Vasiliki Zoi, Evrysthenis Vartholomatos, Vasiliki Galani, Athanassios P. Kyritsis, George A. Alexiou, Diamanto Lazari

**Affiliations:** 1 Neurosurgical Institute, University of Ioannina, 45500 Ioannina, Greece; 2 Laboratory of Pharmacognosy, Division of Pharmacognosy-Pharmacology, School of Pharmacy, Faculty of Health Sciences, Aristotle University of Thessaloniki, 54124 Thessaloniki, Greece; 3 Department of Anatomy Histology-Embryology, School of Medicine, University of Ioannina, 45110 Ioannina, Greece; 4 Department of Neurosurgery, University of Ioannina, 45500 Ioannina, Greece

**Keywords:** siderol, glioblastoma, cell cycle arrest, cell viability, temozolomide

## Abstract

Glioblastoma (GBM) is the most aggressive primary central nervous system (CNS) tumor in adults with dismal prognosis. Currently, the therapeutic interventions include gross total resection, when possible, followed by radiotherapy and chemotherapy. However, despite treatment, tumor usually recurs within 7–9 months. The presence of glioma cells with stem-like properties and tumor’s heterogeneity have been identified as the most important factors driving recurrence. Recently, research efforts have been focused on the use of natural substances as treatment for GBM. Siderol is an ent-kaurane diterpenoid, isolated from the genus *Sideritis*. *Sideritis* extracts have already been investigated for their anti-inflammatory, antioxidant, and anticancer effects. In this study, we investigated the antitumoral effects of siderol in GBM T98 and U87 cell lines, as well as the effects of combined treatment with temozolomide (TMZ). Cell viability was evaluated by the 3-(4,5-dimethylthiazol-2-yl)-2,5-diphenyltetrazolium bromide (MTT) assay and trypan blue exclusion assay. Different concentrations of siderol were used in order to calculate the IC50 values at 72 h after treatment. Flow cytometry used for the DNA cell cycle analysis after treatment with siderol in concentrations of IC50 and twice the IC50 values for 72 h. Furthermore, the effect of siderol in cell’s migratory ability was tested using wound healing assay. Cell viability and proliferation, after combined treatment with siderol and TMZ, also were evaluated with the trypan blue exclusion assay and the effects of the combination treatment were analyzed with CompuSyn software. Treatment with siderol significantly reduced cell viability in T98 and U87 cell lines in a dose-dependent manner and IC50 values were calculated, 18 μM and 13 μM, respectively. Moreover, siderol induced G_0_/G_1_ cell cycle arrest in a dose-dependent manner and inhibited the migration in both cell lines. In addition, siderol and TMZ seem to have synergistic action in the majority of tested concentrations in both T98 and U87 cells. In conclusion, siderol may represent an innovative strategy for the treatment of GBM, and further studies are needed on siderol’s efficacy and mode of action.

## 1. Introduction

GBM is the most aggressive tumor of the CNS with poor clinical prognosis in adults [1]. Currently, the therapeutic interventions include surgery, radiotherapy, and chemotherapy; however, the mean survival period of patients still remains short, ranging from 14 to 16 months—only 9.8% of GBM patients can reach a survival period of up to 5 years [2,3]. Several drugs are FDA approved for GBM, with TMZ being the standard chemotherapy. Nevertheless, TMZ increases the median survival by only 2.5 months, while higher TMZ doses can cause systemic toxicity [4,5]. Additionally, the BBB, which permits the selective penetration of some low molecular weight compounds and blocks several chemotherapeutic medications, prevents the majority of pharmaceuticals from entering the brain [6,7]. Due to the failure of classical chemotherapies and targeted drugs, research efforts have focused on the use of less toxic substances. Therefore, a variety of organic compounds are assessed for their potential to act as GBM therapy agents. These organic substances slow tumor growth and promote GBM cell death [8].

Siderol is an ent-kaurane diterpenoid, isolated from the genus *Sideritis* (species *S. scardica*), also known as “ironwort” and “mountain tea”. *Sideritis* is a genus of flowering plants well known for their use as herbal medicine, especially in Greece, Turkey, Albania, Bulgaria, and other countries. The stems, leaves, and flowers of *S. scardica* are widely decocted or infused to make herbal tea (aerial parts). This herbal medicine has been used for centuries to improve immunity, support the digestive system, and lessen the symptoms of numerous ailments. According to several studies, this plant may have a beneficial impact on many common illnesses, especially when it comes to its anti-microbial, anti-inflammatory, and antioxidant properties. The main active compounds present in this genus are diterpenoids and flavonoids [9,10,11].

As a result of the high concentration of phenolic compounds, *S. scardica* extracts perform dose-dependent anti-inflammatory and gastroprotective activities. The control of pro-inflammatory mediators (NF-B, TNF-, IL-1, and IL-6) is key to the management of inflammation [12,13]. Studies have shown that the ethanolic extract of *S. scardica* has considerable anti-inflammatory properties that are comparable to those of the nonsteroidal anti-inflammatory medication indomethacine. Moreover, several studies have shown that *S. scardica* preparations have antibacterial action against a number of typical Gram-positive and Gram-negative bacteria, as well as the yeast *Candida albicans* [14]. Concerning the antioxidant properties, *Sideritis* extracts have the capacity to prevent reactive oxygen species from damaging DNA, to reduce the amount of damage caused, and to increase the effectiveness of DNA repair mechanisms [15,16].

**Figure 1:**
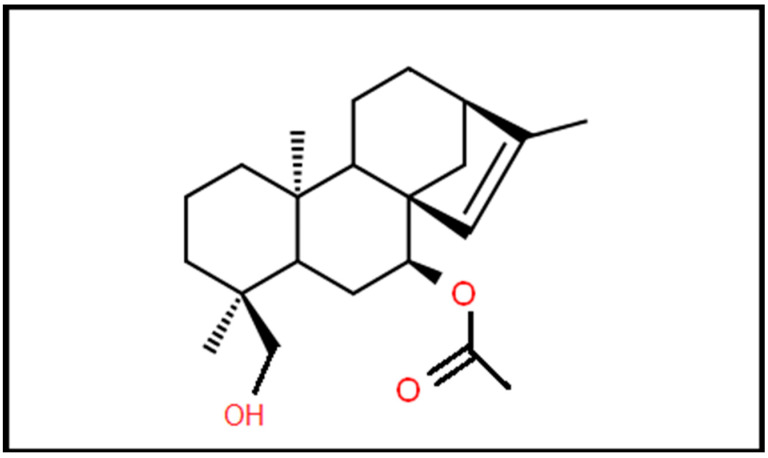
Molecular structure of siderol.

Additionally, the results of certain studies seem to be promising for the use of *Sideritis* extracts as a treatment against different types of cancer such as liver and colon cancer [11]. The high concentration of several phenolic compounds, particularly a few flavonoids, may result in cytotoxic action against cancer cells [14]. Furthermore, the molecular weight of siderol is 346.5 Daltons that permit this agent to cross the BBB (Figure 1). In this study, we investigated the antitumoral effects of Siderol in U87 and T98 GBM cell lines, alone and in combination with TMZ.

## 2. Materials and Methods

### 2.1. Cell Lines and Conditions for Treatment

The human glioma cell lines U87 and T98 were provided by Dr W.K. Alfred Yung (Department of Neuro-Oncology, M.D. Anderson Cancer Center, Houston, TX, USA) and ATCC (Manassas, VA, USA), respectively. Both cell lines were grown in DMEM (Gibco BRL, Life Technologies, Grand Island, NY, USA), after being enriched with 10% FBS and 1% penicillin-streptomycin (Gibco BRL). The cell’s incubation carried out in humidified conditions containing 5% CO_2_ at 37 °C. Siderol isolated from the hexane extract of cultivated *Sideritis scardica* diluted in DMSO and storage at −80 °C. TMZ was obtained from Sigma Aldrich, diluted in DMSO and storage at −20 °C. Before every experiment, siderol and TMZ were diluted from stock solution to the final concentration with DMEM.

### 2.2. Viability Assay

Cell viability was evaluated by the MTT (Sigma Life Sciences, Grand Island, NY, USA) assay and trypan blue exclusion assay. Approximately 5000 cells were seeded in 96-well plates, and after 24 h were treated with siderol in concentrations of 10–300 μM. At 72 h, after siderol treatment, MTT was added. The MTT-formazan concentration was measured at 570 nm. For the trypan blue exclusion assay, 20,000 cells were seeded in 12-well plates and were incubated for 24 h. Afterward, they were treated with siderol in concentrations of 10–60 μM for 72 h. Trypan blue exclusion assay was carried out. At least three times each of both approaches were used, and the results were expressed as the mean of the three. Cell viability after treatment with TMZ, in both cell lines, was also evaluated by the trypan blue exclusion assay. T98 cells were treated with TMZ in concentrations of 100–600 μM for 72 h and U87 cells in concentrations 20–100 μM for 72 h.

### 2.3. Flow Cytometric Analysis of DNA Cell Cycle

For the DNA cell cycle analysis, 20,000 cells were seeded in 12-well plates, and after 24 h were treated with siderol in concentrations of IC50 and twice the IC50 values for another 72 h. Subsequently, cells were washed with PBS solution, harvested after incubation with trypsin, and held at 37 °C for 20 min with a PI working solution (50 g/mL PI, 20 mg/mL RNase A, and 0.1% Triton X-100). PI fluorescence data were collected using a flow cytometer (Omnicyt, Cytognos S.L., Grand Island, NY, USA) and were analyzed using the GraphPad Prism version 6 software and MedCalc software (Trial version).

### 2.4. Wound Healing Assay

In 6-well plates, cells (10⁵) were plated and incubated until they achieved 70–80% confluence. Afterward, DMEM rejected, the monolayer cells were scratched using a 200 μL pipette tip at the bottom of the well and were cultivated under standard conditions in DMEM supplement with 1% FBS, after being treated with siderol at IC50 and half IC50 concentrations. Each cell line’s migratory distance was evaluated at 0, 24, and 48 h after scratching using the ImageJ software. Data were analyzed using the GraphPad Prism version 6 software.

### 2.5. Combination Treatment with Siderol and TMZ

To evaluate if there is a synergistic action in combination of siderol and TMZ, T98 and U87 cells were treated with siderol, TMZ and different combinations of them. Cells were cultured in 24-well plates, and after 24 h were treated with siderol, TMZ and their combination in five different concentrations between half and twice IC50. T98 cells were treated with siderol in concentrations 9 μM, 13.5 μM, 18 μM, 27 μM, and 36 μM while with TMZ in concentrations 165 μM, 247 μM, 330 μM, 495 μM, and 660 μM. U87 cells were treated with siderol in concentrations 6.5 μM, 9.75 μM, 13 μM, 19.5 μM, and 26 μM, and with TMZ in concentrations 25 μM, 37.5 μM, 50 μM, 75 μM, and 100 μM. The substances’ concentrations combined proportional from lowest to highest. After 72 h, viability was calculated using the trypan blue exclusion assay.

### 2.6. Statistical Analysis

The mean and SD were used to express the data. We employed the *t*-test for multiple comparisons to examine the significance of differences between the outcomes of several experimental conditions. A difference was deemed significant when the *p* value was less than 0.05.

## 3. Results

### 3.1. Viability of GBM Cells after Treatment with Siderol and IC50 Calculation

**Figure 2:**
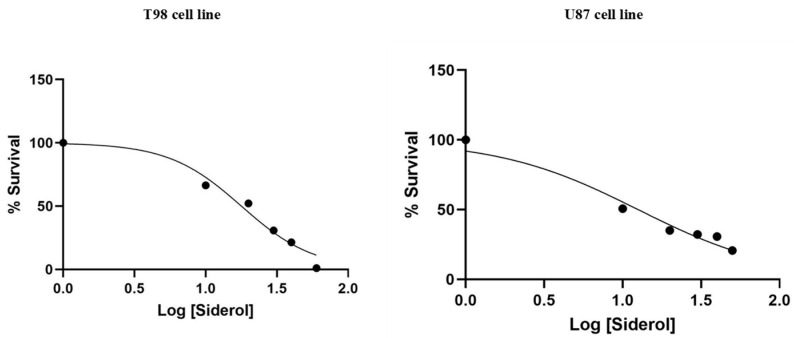
Cytotoxic effect of Siderol on T98 and U87 cell lines, 72 h after treatment. Values are the means of three independent experiments and are normalized to non-treated cells. The IC50 values were determined using the non-linear regression analysis model of GraphPad Prism Version 6.

**Figure 3:**
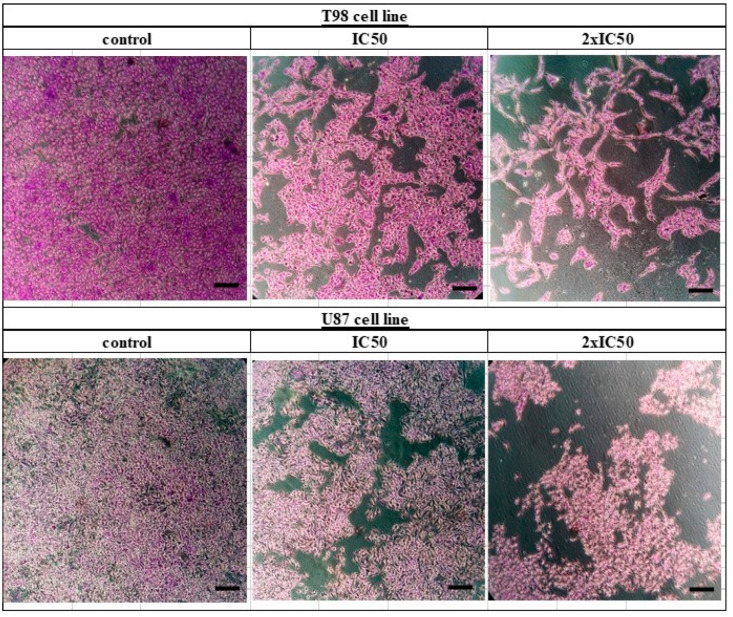
Crystal violet staining (0.2% Crystal Violet) of T98 and U87 cell lines. Cells were seeded in 6-well plates and after 24 h were treated with siderol in concentrations of IC50 and twice IC50 values. Crystal violet solution was added 72 h later. Images were recorded at 10× magnification. Scales bars = 100 μM.

T98 and U87 cells were cultured with escalating siderol concentrations for 72 h to test the susceptibility of GBM cells to siderol. Both cell lines responded to siderol therapy with varying degrees of sensitivity. In T98 cells and U87 cells, siderol’s IC50 value for reduced viability was 18 μM and 13 μM, respectively (Figure 2). When siderol concentrations were increased, cells underwent changes such as cell shrinkage and death, which could be seen under a microscope (Figure 3).

### 3.2. Viability of GBM Cells after Treatment with TMZ and IC50 Calculation

**Figure 4:**
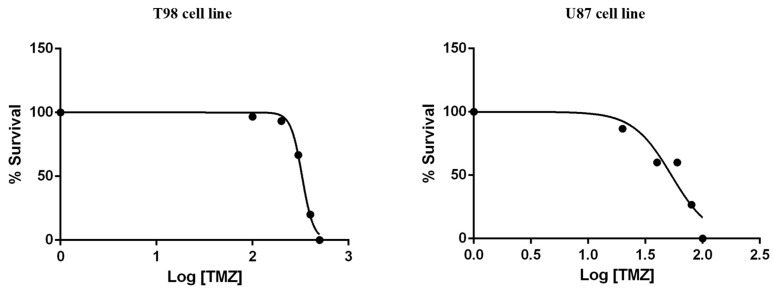
Cytotoxic effect of TMZ on T98 and U87 cell lines, 72 h after treatment. Values represent the means of three independent experiments and are normalized to non-treated cells. The non-linear regression analysis methodology of GraphPad Prism Version 6 was used to determine the IC50 values.

To calculate the IC50 values for TMZ, T98 and U87 cells were treated with increasing TMZ concentrations for 72 h. The IC50 values of reduced viability for TMZ were 330 μM in T98 cells and 50 μM in U87 cells (Figure 4). 

### 3.3. Siderol Induced G_0_/G_1_ Cell Cycle Arrest in Both Cell Lines

**Figure 5:**
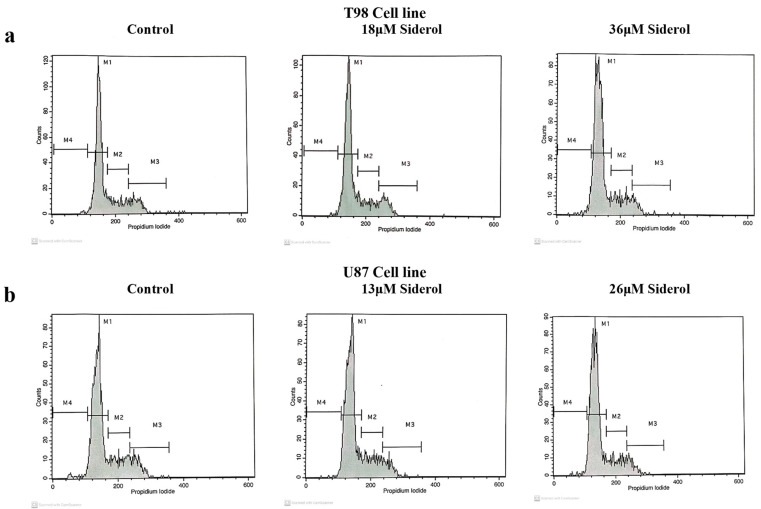
Histogram representation of cell-cycle distribution in T98 (**a**) and U87 (**b**) cell lines after treatment with siderol in concentrations of IC50 and twice IC50 values.

**Figure 6:**
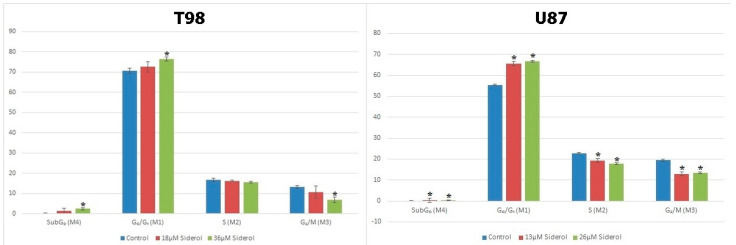
Graphical representation of cell-cycle distribution in both cell lines after treatment with siderol in IC50 and twice IC50 concentrations. An asterisk is used to indicate differences that were significant (*p* < 0.05).

To investigate the effects of siderol on cell cycle progression in T98 and U87 cell lines, cells were treated with IC50 and twice IC50 values of siderol for 72 h. Siderol induced G_0_/G_1_ cell cycle arrest in both cell lines in a dose-dependent manner (Figure 5 and Figure 6, Table 1).

### 3.4. Siderol Inhibited Cell Migration in Both Cell Lines

**Figure 7:**
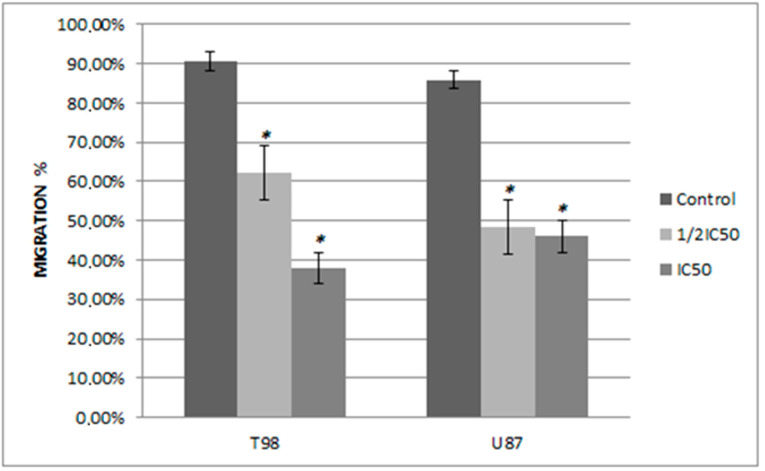
The effect of siderol on the migratory ability of T98 and U87 GBM cell lines at 48 h. Width_migration_ = Width_0h_ − Width_48h_. The experiment was carried out three times. Values expressed as percentages of migration and at 0 h the wound widths were set to 0%. An asterisk is used to indicate differences that are significant (*p* < 0.05).

**Figure 8:**
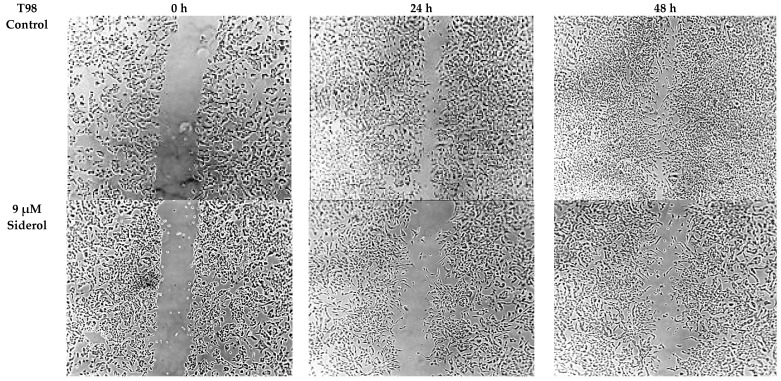

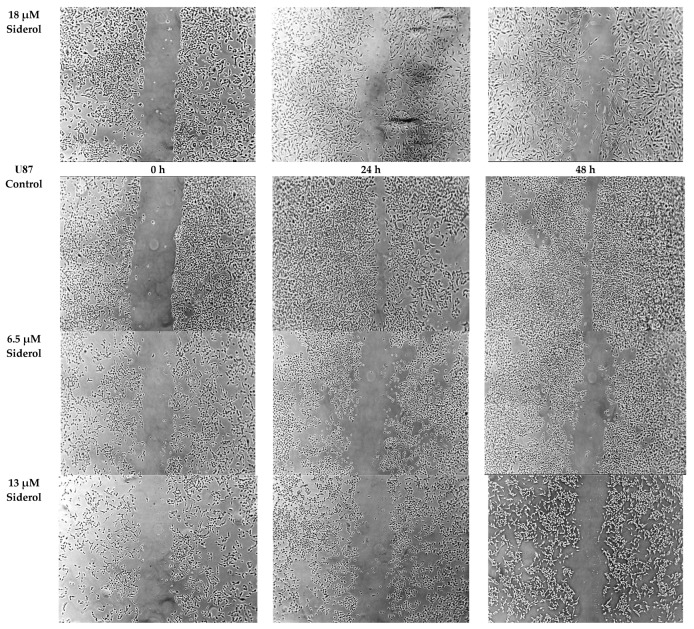
The effect of siderol on the migratory ability of T98 and U87 GBM cell lines at 0, 24, and 48 h, after treatment with IC50 and half IC50 values. Images were recorded at 5× magnification. Scale bars = 200 μM.

In order to determine if siderol could influence the migration of the T98 and U87 cell lines into a scratch-induced wound, a cell monolayer demonstrated that siderol strongly hindered both cell lines’ ability to heal their wounds at concentrations of IC50 and half IC50 values (Figure 7 and Figure 8).

### 3.5. Siderol and TMZ Have Synergistic Effect in Both Cell Lines

**Figure 9:**
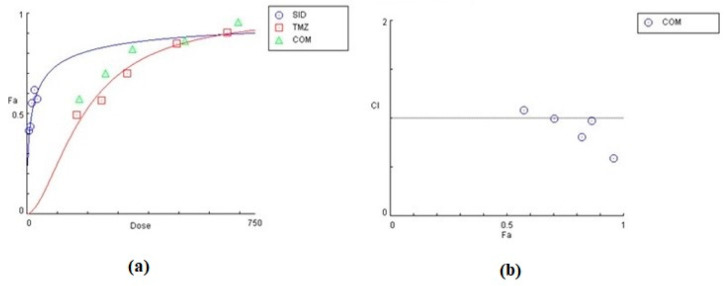
Graphical representation of the combinatorial effect of siderol and TMZ in T98 cell line, from CompuSyn Report. The dose-effect curve (**a**) for each substance, as for the combination of them. The *x*-axis represents the concentrations of substances and the *y*-axis the effect (% mortality). The Combination Index plot (**b**) represents the combinations where CI < 1, =1 or >1.

**Figure 10:**
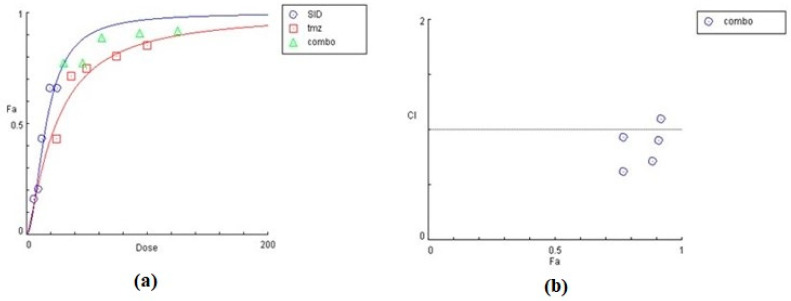
Graphical representation of the combinatorial effect of siderol and TMZ in U87 cell line, from CompuSyn Report. The dose-effect curve (**a**) for each substance, as for the combination of them. The *x*-axis represents the concentrations of substances and the *y*-axis the effect (% mortality). The Combination Index plot (**b**) represents the combinations where CI < 1, =1, or >1.

The effect of siderol in combination with TMZ, in both cell lines, is summarized in Table 2 and Table 3, as the concentrations of each substance combined. In both cell lines, siderol and TMZ have synergistic effect in the majority of tested combinations. In T98 cells, the highest synergism was monitored when siderol and TMZ were given between half IC50 and IC50 values (13.5 μM Siderol and 247 μM TMZ), while antagonistic effect was monitored only at the lowest concentrations. Contrariwise, in U87 cells, antagonistic behavior showed up only at the highest concentrations (Figure 9 and Figure 10). However, further studies are required.

## 4. Discussion

GBM is the most aggressive primary brain tumor. Surgery, radiotherapy, and chemotherapy are the most common treatments against GBM, but the mean survival period still remains short [3,17,18]. In addition, both radio and chemo resistance constitute inhibitory factors for the successful treatment of GBM [19]. Natural compounds constitute an important research field since most of these substances are of low toxicity [5]. Novel products from natural compounds protect glial cells by decreasing oxidative stress and neuroinflammation. They also restrain proliferation, activating apoptosis pathways and inhibiting pro-oncogene events [20,21]. These products could contribute to the development of new therapeutic options that improve patient quality of life and extend survival [22]. In addition, many natural substances have been investigated for their synergistic effect with TMZ or/and radiation and the results seem to be encouraging [23,24].

Siderol is an ent-kaurane diterpenoid obtained from the hexane extract of cultivated *Sideritis scardica* (Lamiaceae) [25]. *Sideritis* involves around 150 species, of which many are popular herbal medicines in Mediterranean folk medicine [26,27]. “Ironwort” is the common name for the aerial parts of various species of *Sideritis* plants. According to EMA, ironwort preparations can be used for the relief of cough associated with a cold and for the relief of mild stomach and gut discomfort. Furthermore, there is evidence that they have been used safely for at least 30 years, although there is insufficient evidence from clinical trials.

In previous studies, *Sideritis* extracts have been investigated for their interesting biological activities, such as antioxidant, anti-inflammatory, anti-neuropathic, and antimicrobial properties [28,29,30,31]. Their ability to safeguard the DNA from reactive oxygen species, to curtail the inflicted damage and improve the efficiency of the DNA repair mechanisms, has been evaluated [15,16]. Moreover, plants of this genus, that are rich in polyphenolic compounds, such as flavonoids, can produce a therapeutic effect by reducing the symptoms of inflammatory processes. The management of inflammation and neuropathic pain mainly succeed by the management of pro-inflammatory mediators (NF-κB, TNF-α, IL-1β, and IL-6) [12,13]. Other studies have demonstrated the anti-aging effect of *Sideritis* extracts, by anti-hyaluronidase activity [32,33] and the antimicrobial activities [34,35].

Furthermore, several studies have focused on the anti-cancer activity of *Sideritis* extracts. DLD1 (human colon adenocarcinoma), HL60 (human promyelocytic leukemia), and ARH77 (human myeloma) cell lines, have been investigated, after treatment with methanolic extracts of *Sideritis* species. The data revealed that the used extracts had cytotoxic activity against all applied cancer cell types in a dose and time-dependent manner. The cell death occurs by apoptosis. The effect of *Sideritis* extracts on the expression level of the pro-apoptotic gene *CASPASE 3*, evaluated and found to have positive effect [36,37,38]. Other genes, which are involved at the apoptotic mechanism in cells, such as *BAX*, *BCL2*, *p53,* and *TNF* have also been investigated in DLD1 cell lines and the results confirmed that *Sideritis* extracts lead cells to apoptosis [11]. Additionally, in MCF-7 (human breast cancer) cell line, cell proliferation and viability reduction was demonstrated after treatment with *Sideritis* methanol extracts [39].

In this study, we investigated the cytotoxic effect of siderol, an ent-kaurane diterpenoid isolated from the hexane extract of cultivated *Sideritis scardica*, in GBM cell lines. The results determine that siderol caused a significant cell viability and proliferation inhibition in a dose-dependent manner, and the IC50 values in both applied cell lines are relatively low. Furthermore, siderol induced G_0_/G_1_ phase cell cycle arrest in both cell lines. These results confirm the cell proliferation inhibition by blocking the cell cycle development [40,41].

Another important result of our research is that siderol inhibited the cell migration. The migratory ability of cancer cells constitutes an important failure reason in GBM treatments. Even after a successful surgery, cancer cells, which have already migrated, can recreate a new tumor [42]. Siderol inhibited the migratory ability of T98 and U87 cell lines in a dose and time-dependent manner and there are significant differences compared to controls.

Concerning the combined treatment of siderol and TMZ, the experiments proved a synergistic effect in both cell lines. T98 cells are resistant to TMZ and high concentration for treatment is required. Nevertheless, higher doses of TMZ may cause higher risk of adverse effects. The synergistic effect with siderol may be a possible solution using lower doses of TMZ and/or overcoming the resistance of cells. Summarizing, the results of combined treatment may be promising at GBM treatment as the TMZ’s dose could be decreased. Therefore, further experiments are needed to fully determine the synergy between siderol and TMZ in GBM cells.

The present study has several limitations. Siderol’s exact mechanism of action should be investigated, as well as the synergistic mechanism with TMZ. In addition, siderol should be evaluated with in vivo models to determine if there is toxicity [43,44]. Proliferation data can be further verified using other methods such as clonogenic assay, cell-cycle analysis, or mRNA/protein expression analysis of proliferative markers. The effect of siderol and temozolomide, alone and in combination, on cell apoptosis should be also investigated. Concerning the synergistic action, siderol could be examined in combination with radiation, as, today, radiotherapy and chemotherapy are the standard therapeutic interventions in GBM treatment. Furthermore, the investigation of siderol’s action on other GBM cell lines would be a contribution to determine the further possible effects of siderol.

The development of new natural products as a treatment for GBM constitute important goal for researchers, as these products reduce the probability of toxicity, which other chemotherapy drugs bring out. Siderol may represent an innovative strategy for the treatment of GBM.

## Figures and Tables

**Table 1:** Cell-cycle distribution assessed by flow cytometry in T98 (**a**) and U87 (**b**) glioblastoma cells after treatment with siderol. The experiment was carried out three times and the values are means of three repetitions. Significant differences (*p* < 0.05) are marked with an asterisk.

**a**	**Treatment**	**SubG₀ (M4)**	**G₀/G₁ (M1)**	**S (M2)**	**G₂/M (M3)**
	Control	0.26 ± 0.1	70.64 ± 1.3	16.58 ± 0.8	13.19 ± 0.7
	18 μM Siderol	1.3 ± 1.2	72.5 ± 2.5	16.24 ± 0.4	10.6 ± 3.1
	36 μM Siderol	2.4 ± 0.6 *	76.37 ± 1.1 *	15.57 ± 0.4	6.86 ± 1.2 *
**b**	**Treatment**	**SubG₀ (M4)**	**G₀/G₁ (M1)**	**S (M2)**	**G₂/M (M3)**
	Control	0 ± 0.01	55.4 ± 0.5	22.7 ± 0.4	19.4 ± 0.7
	13 μM Siderol	0.23 ± 0.03 *	65.47 ± 0.59 *	19.13 ± 0.74 *	12.97 ± 0.12 *
	26 μM Siderol	0.3 ± 0.15 *	66.7 ± 0.5 *	17.8 ± 0.44 *	13.5 ± 0.44 *

**Table 2:** Assessment of combinatorial effect of siderol and TMZ in T98 cells. Concentrations of each substance, the effect (% mortality), and CI of each combination are represented. Data were determined by CompuSyn software. CI allows quantitative determination of drug interactions, where CI < 1, =1 and >1 indicates synergism, additive effect and antagonism, respectively.

Siderol (μM)	Temozolomide (μM)	Effect	CI	Conclusion
9	165	0.57	1.08150	Antagonism
13.5	247	0.70	0.99326	Synergism
18	330	0.82	0.80500	Synergism
27	495	0.86	0.97489	Synergism
36	660	0.96	0.58721	Synergism

**Table 3:** Assessment of combinatorial effect of siderol and TMZ in U87 cells. Concentrations of each substance, the effect (% mortality) and CI of each combination are represented. Data were determined by CompuSyn software. CI allows quantitative determination of drug interactions, where CI < 1, =1 and >1 indicates synergism, additive effect and antagonism, respectively.

Siderol (μM)	Temozolomide (μM)	Effect	CI	Conclusion
6.5	25	0.77	0.61869	Synergism
9.75	37.5	0.77	0.92803	Synergism
13	50	0.89	0.71018	Synergism
19.5	75	0.91	0.90397	Synergism
26	100	0.92	1.0944	Antagonism

## Abbreviations

GBM	Glioblastoma
CNS	Central Nervous System
TMZ	Temozolomide
MTT	3-(4,5-dimethylthiazol-2-yl)-2,5-diphenyltetrazolium bromide
FDA	Food and Drug Administration
BBB	Blood–Brain Barrier
DMEM	Dulbecco’s Modified Eagle’s Medium
DMSO	Dimethyl Sulfoxide
PBS	Phosphate-Buffered Saline
PI	Propidium Iodide
FBS	Fetal Bovine Serum
SD	Standard Deviation
CI	Combination Index
EMA	European Medicines Agency

## References

[1] Weathers S.P., Gilbert M.R. (2014). Advances in treating glioblastoma. F1000Prime Rep..

[2] Dorte S.N., Poulsen H., Lassen U. (2016). Hallmarks of glioblastoma: A systematic review. ESMO Open.

[3] Stupp R., Mason W.P., van den Bent M.J., Weller M., Fisher B., Taphoorn M.J.B., Belanger K., Brandes A.A., Marosi C., Bogdahn U. (2005). Radiotherapy plus concomitant and adjuvant temozolomide for glioblastoma. N. Engl. J. Med..

[4] Gilbert M.R., Wang M., Aldape K.D., Stupp R., Hegi M.E., Jaeckle K.A., Armstrong T.S., Wefel J.S., Won M., Blumenthal D.T. (2013). Dose-dense temozolomide for newly diagnosed glioblastoma: A randomized phase III clinical trial. J. Clin. Oncol..

[5] Vengoji R., Macha M.A., Batra S.K., Shonka N.A. (2018). Natural products: A hope for glioblastoma patients. Oncotarget.

[6] Levin V.A., Ellingson B.M. (2018). Understanding brain penetrance of anticancer drugs. Neuro. Oncol..

[7] Banks W.A. (2009). Characteristics of compounds that cross the blood-brain barrier. BMC Neurol..

[8] Zhai K., Siddiqui M., Abdellatif B., Liskova A., Kubatka P., Büsselberg D. (2021). Natural Compounds in Glioblastoma Therapy: Preclinical Insights, Mechanistic Pathways, and Outlook. Cancers.

[9] Sarikurkcu C., Locatelli M., Mocan A., Zengin G., Kirkan B. (2020). Phenolic Profile and Bioactivities of Sideritis perfoliata L.: The Plant, Its Most Active Extract, and Its Broad Biological Properties. Front. Pharmacol..

[10] Axiotis E., Petrakis E.A., Halabalaki M., Mitakou S. (2020). Phytochemical Profile and Biological Activity of Endemic Sideritis sipylea Boiss. in North Aegean Greek Islands. Molecules.

[11] Şimşek E., Uysal T. (2018). The Effects of the Sideritis ozturkii Extract on the Expression Levels of some Apoptotic Genes. Curr. Perspect. Med. Aromat. Plants (CUPMAP).

[12] González-Burgos E., Carretero M.E., Gómez-Serranillos M.P. (2011). Sideritis spp.: Uses, chemical composition and pharmacological activities—A review. J. Ethnopharmacol..

[13] Cavalcanti M.R.M., Passos F.R.S., Monteiro B.S., Gandhi S.R., Heimfarth L., Lima B.S., Nascimento Y.M., Duarte M.C., Araujo A.A., Menezes I.R. (2021). HPLC-DAD-UV analysis, anti-inflammatory and anti-neuropathic effects of methanolic extract of Sideritis bilgeriana (lamiaceae) by NF-κB, TNF-α, IL-1β and IL-6 involvement. J. Ethnopharmacol..

[14] Żyżelewicz D., Kulbat-Warycha K., Oracz J., Żyżelewicz K. (2020). Polyphenols and Other Bioactive Compounds of Sideritis Plants and Their Potential Biological Activity. Molecules.

[15] Oalđe M., Kolarević S., Živković J., Aradski A.A., Marić J.J., Kolarević M.K., Đorđević J., Marin P.D., Šavikin K., Vuković-Gačić B. (2021). A comprehensive assessment of the chemical composition, antioxidant, genoprotective and antigenotoxic activities of Lamiaceae species using different experimental models in vitro. Food Funct..

[16] Stagos D., Portesis N., Spanou C., Mossialos D., Aligiannis N., Chaita E., Panagoulis C., Reri E., Skaltsounis L., Tsatsakis A.M. (2012). Correlation of total polyphenolic content with antioxidant and antibacterial activity of 24 extracts from Greek domestic Lamiaceae species. Food Chem. Toxicol..

[17] Ma R., Taphoorn M.J.B., Plaha P. (2021). Advances in the management of glioblastoma. J. Neurol. Neurosurg. Psychiatry.

[18] Liu S., Shi W., Zhao Q., Zheng Z., Liu Z., Meng L., Dong L., Jiang X. (2021). Progress and prospect in tumor treating fields treatment of glioblastoma. Biomed. Pharmacother..

[19] Goenka A., Tiek D., Song X., Huang T., Hu B., Cheng S.Y. (2021). The Many Facets of Therapy Resistance and Tumor Recurrence in Glioblastoma. Cells.

[20] Uddin M.S., Kabir M.T., Mamun A.A., Sarwar S., Nasrin F., Bin Emran T., Alanazi I.S., Rauf A., Albadrani G.M., Sayed A.A. (2021). Natural Small Molecules Targeting NF-κB Signaling in Glioblastoma. Front. Pharmacol..

[21] Soukhtanloo M., Mohtashami E., Maghrouni A., Mollazadeh H., Mousavi S.H., Roshan M.K., Tabatabaeizadeh S.-A., Hosseini A., Vahedi M.M., Jalili-Nik M. (2020). Natural products as promising targets in glioblastoma multiforme: A focus on NF-κB signaling pathway. Pharmacol. Rep..

[22] Behl T., Sharma A., Sharma L., Sehgal A., Singh S., Sharma N., Zengin G., Bungau S., Toma M., Gitea D. (2021). Current Perspective on the Natural Compounds and Drug Delivery Techniques in Glioblastoma Multiforme. Cancers.

[23] Arcella A., Sanchez M. (2021). Natural substances to potentiate canonical glioblastoma chemotherapy. J. Chemother..

[24] Zoi V., Galani V., Vartholomatos E., Zacharopoulou N., Tsoumeleka E., Gkizas G., Bozios G., Tsekeris P., Chousidis I., Leonardos I. (2021). Curcumin and Radiotherapy Exert Synergistic Anti-Glioma Effect In Vitro. Biomedicines.

[25] Koromili M., Kapourani A., Koletti A., Papandreou G., Assimopoulou A.N., Lazari D., Barmpalexis P. (2022). Preparation and Evaluation of Siderol Amorphous Solid Dispersions: Selection of Suitable Matrix/Carrier. AAPS PharmSciTech.

[26] Pihan L.A., Peter S., Vollmer G., Meier B., Wolfram E. (2021). HPTC Fingerprint Authentication of Selected Sideritis spp. Using a Pharmacognostic Approach. Planta Med..

[27] Romanucci V., Di Fabio G., D′Alonzo D., Guaragna A., Scapagnini G., Zarrelli A. (2017). Traditional uses, chemical composition and biological activities of Sideritis raeseri Boiss. & Heldr. J. Sci. Food Agric..

[28] Çelik T., Önderci M., Pehlivan M., Yumrutaş Ö., Üçkardeş F. (2021). In vitro scolicidal effects of Sideritis perfoliata extract against Echinococcus granulosus. Int. J. Clin. Pract..

[29] Güvenç A., Okada Y., Akkol E.K., Duman H., Okuyama T., Çalış I. (2010). Investigations of anti-inflammatory, antinociceptive, antioxidant and aldose reductase inhibitory activities of phenolic compounds from Sideritis brevibracteata. Food Chem..

[30] Tomou E.M., Lytra K., Chrysargyris A., Christofi M.-D., Miltiadous P., Corongiu G.L., Tziouvelis M., Tzortzakis N., Skaltsa H. (2022). Polar constituents, biological effects and nutritional value of Sideritis sipylea Boiss. Nat. Prod. Res..

[31] Charami M.T., Lazari D., Karioti A., Skaltsa H., Hadjipavlou-Litina D., Souleles C. (2008). Antioxidant and antiinflammatory activities of Sideritis perfoliata subsp. perfoliata (Lamiaceae). Phytother. Res..

[32] Sklirou A.D., Angelopoulou M.T., Argyropoulou A., Chaita E., Boka V., Cheimonidi C., Niforou K., Mavrogonatou E., Pratsinis H., Kalpoutzakis E. (2021). Phytochemical Study and In Vitro Screening Focusing on the Anti-Aging Features of Various Plants of the Greek Flora. Antioxidants.

[33] Tomou E.-M., Papaemmanouil C.D., Diamantis D.A., Kostagianni A.D., Chatzopoulou P., Mavromoustakos T., Tzakos A.G., Skaltsa H. (2021). Anti-Ageing Potential of S. euboea Heldr. Phenolics. Molecules.

[34] Loğoğlu E., Arslan S., Oktemer A., Sakõyan I. (2006). Biological activities of some natural compounds from Sideritis sipylea Boiss. Phytother. Res..

[35] Aligiannis N., Kalpoutzakis E., Chinou I.B., Mitakou S., Gikas E., Tsarbopoulos A. (2001). Composition and antimicrobial activity of the essential oils of five taxa of Sideritis from Greece. J. Agric. Food Chem..

[36] Şimşek Sezer E.N., Uysal T. (2021). Phytochemical Analysis, Antioxidant and Anticancer Potential of Sideritis niveotomentosa: Endemic Wild Species of Turkey. Molecules.

[37] Demirelma H., Gelinci E. (2019). Determination of the Cytotoxic Effect on Human Colon Cancer and Phenolic Substance Content of the Endemic Species Sideritis Ozturkii Aytaç & Aksoy. Appl. Ecol. Environ. Res..

[38] Porter A., Jänicke R. (1999). Emerging roles of caspase-3 in apoptosis. Cell Death Differ..

[39] Yumrutas O., Oztuzcu S., Ozturk N., Pehlivan M., Poyraz E., Bozgeyik I., ZiyaIÄŸci Y., Cevik M.O., Aksoy A.F., BagÄ±s H. (2015). Cell viability, anti-proliferation and antioxidant activities of Sideritis syriaca, Tanacetum argenteum subsp. argenteum and Achillea aleppica subsp. zederbaueri on human breast cancer cell line (MCF-7). J. App. Pharm. Sci..

[40] Wang R., Liu T., Chen J., Zhang D. (2022). Paradol Induces Cell Cycle Arrest and Apoptosis in Glioblastoma Cells. Nutr. Cancer.

[41] Chen J., Ouyang Y., Cao L., Zhu W., Zhou Y., Zhou Y., Zhang H., Yang X., Mao L., Lin S. (2013). Diazepam inhibits proliferation of human glioblastoma cells through triggering a G_0_/G_1_ cell cycle arrest. J. Neurosurg. Anesthesiol..

[42] Liu X., Chen J.Y., Chien Y., Yang Y.P., Chen M.T., Lin L.T. (2021). Overview of the molecular mechanisms of migration and invasion in glioblastoma multiforme. J. Chin. Med. Assoc..

[43] Tomou E.-M., Chatziathanasiadou M.V., Chatzopoulou P., Tzakos A.G., Skaltsa H. (2020). NMR-Based Chemical Profiling, Isolation and Evaluation of the Cytotoxic Potential of the Diterpenoid Siderol from Cultivated Sideritis euboea Heldr. Molecules.

[44] Lytra K., Tomou E.M., Chrysargyris A., Christofi M., Miltiadous P., Tzortzakis N., Skaltsa H. (2021). Bio-Guided Investigation of Sideritis cypria Methanol Extract Driven by in Vitro Antioxidant and Cytotoxic Assays. Chem. Biodivers..

